# Geographic variation and associated factors of long-acting contraceptive use among reproductive-age women in Ethiopia: a multi-level and spatial analysis of Ethiopian Demographic and Health Survey 2016 data

**DOI:** 10.1186/s12978-021-01171-2

**Published:** 2021-06-10

**Authors:** Oumer Abdulkadir Ebrahim, Ejigu Gebeye Zeleke, Atalay Goshu Muluneh

**Affiliations:** 1 Department of Public Health, College of Health Sciences, Samara University, Samara, Ethiopia; 2 Department of Epidemiology and Biostatistics, College of Medicine and Health Sciences, University of Gondar, P.O. Box 196, Gondar, Ethiopia

**Keywords:** Long-acting contraceptive, Geographic variation, Ethiopia

## Abstract

**Background:**

High fertility rates and unintended pregnancies are public health concerns of lower and middle income countries such as Ethiopia. Long acting contraceptives (LACs) take the lion’s share in reducing unintended pregnancies and high fertility rates. Despite their numerous advantages, the utilization of LACs remains low in Ethiopia. This study is aimed to explore the geographic variation and associated factors of long acting contraceptive use among reproductive-age women in Ethiopia.

**Methods:**

This is a secondary data analysis of 2016 Ethiopian Demographic and Health Survey (EDHS) data. A total of weighted sample sizes of 10,439 reproductive-age women were included in the final analysis. To clean and analyze the none-spatial data Stata 14 was used while ArcGIS 10.6 and SaTScanTM version 9.6 software were used for spatial analysis. Multilevel Mixed-effect Logistic regression model was used to identify associated factors of LACs utilization. An Adjusted Odds Ratio (AOR) with 95% Confidence Interval (CI) was reported to identify significant variables.

**Results:**

Long acting contraceptive utilization was non-random (Moran’s I: 0.30, p-value < 0.01). Statistically, clusters with significant low utilization of LACs were found in Somali, Afar, Gambela, northern Amhara, eastern Oromia and western part of Southern Nations Nationalities and Peoples (SNNP) regions. Adjusting for other factors such as being married (AOR = 2.51, 95% CI: 1.29–4.87), having one to two (AOR = 2.14, 95% CI: 1.43–3.22), and three to four children (AOR = 1.68, 95% CI: 1.02–2.76), urban (AOR = 1.59, 95% CI: 1.16–2.19), want no more children (AOR = 1.40, 95% CI: 1.08–1.83), working status of women (AOR = 1.33, 95% CI: 1.07–1.65) increased the odds of LACs utilization. While previous history of abortion (AOR = 0.56, 95% CI: 0.39–0.80), and living in the pastoralist community (AOR = 0.22, 95% CI: 0.14–0.35) reduced the odds of LACs utilization in Ethiopia

**Conclusions:**

Significant geographic variation of LACs utilization was observed in Ethiopia. Spots with Low LACs utilization were found in the eastern, north eastern and western part of the country. Socio-demographic and pregnancy related factors were significant determinants of LACs utilization. Designing intervention programs targeting the identified hot spot clusters, and variables that can hinder the utilization of LACs is very important to increase the utilization.

**Supplementary Information:**

The online version contains supplementary material available at 10.1186/s12978-021-01171-2.

## Plain language summary

Lower and middle-income countries such as Ethiopia face a challenge of an ever increasing population with high maternal mortality. In Ethiopia, the population is estimated to be more than 110 million. High fertility rates, unintended pregnancy, maternal and child mortalities are the main concerns of the country. Accordingly, it is required of the country to make a robust intervention to limit these public concerns. In relation to this, LACs utilization happens to be one of the solutions to solving the concerns. Despite their efficacy, availability, and acceptability the utilization of LACs remains low and varies among different geographic areas. However, the reason is still undefined and geographic variation was not assessed before.

In our study, we analyze the Ethiopian demographic and health survey 2016 data to assess the presence of significant geographic variation and associated factors of long-acting contraceptive utilization. Hence, a spatial and multilevel analysis were employed to assess the geographic variation and associated factors of LACs utilization in Ethiopia.

A statistically significant geographic variation was observed among different clusters. Clusters with significantly low utilization of LACs were found in the pastoralist (Afar, Gambela, and Somalia) regions of the country. Thus, more organized efforts need to be made to increase the utilization of LACs. Controlling for others: Marital statuses, occupation, future pregnancy interest, urban residence, previous history of abortion, living in the pastoralist community were statistically significant determinant factors of LACs utilization.

In conclusion, significant geographic variation of LACs utilization was observed among different clusters. Besides, different socio-demographic, pregnancy, and child health-related variables were significant determinants of LACs utilization.

**Supplementary Information:**

The online version contains supplementary material available at 10.1186/s12978-021-01171-2.

## Background

Long-acting contraceptives (LACs) are a convenient and highly effective modern family planning methods that can avert pregnancy and considerably save costs for couples and governments [1, 2]. Intrauterine device (IUD) and Implants are reversible LACs methods that can avoid pregnancies for a minimum of 3 years while male and female sterilizations are permanent and irreversible methods that can avoid pregnancy forever [3]. Globally, 46 million induced abortions occur each year; and 78,000 maternal deaths occur due to unsafe abortion [4]. In that view, enhancing family planning services is a cost-effective program to avoid unintended pregnancies and decrease maternal and child mortalities [5, 6]. Evidences showed that 44% of maternal mortality can be averted by the provision of contraceptives [5]; however, 225 million women who need to prevent pregnancy are not using convenient and effective family planning methods [7–10]. Globally, 64% of reproductive age women use various forms of family planning methods, of which 34% were long-acting contraceptive methods and more commonly used in Asia and Northern America in 2015. However, the LACs consumption was low in sub-Saharan Africa which accounted for 5.1% [11]. In Ethiopia utilization of family planning is dominated by short-acting methods, and only 10% of married women use LACs [12].

In order to address the unmet need for family planning, the Federal Ministry of Health of Ethiopia (FMOH) has developed a plan to expand contraceptive methods by providing LACs in order to prevent unintended pregnancy at all health facility levels [13]. Despite its numerous advantages, and service accessibility, the utilization of the LACs method was low [1, 6, 12].

Previously, studies done in Ethiopia focused mainly on the prevalence and factors associated with the utilization of long-acting contraceptives. These factors were the age of the women [14, 15], women’s level of education, marital status [16, 17], mass media exposure, wealth index [17, 18], number of live children [17, 19–21] and residence [18, 22]. The finding of these previous studies were focused on the effects of individual-level factors, yet failed to capture in terms geographic variations and the effects of contextual factors.

## Methods

### Study setting, data source and measurement

**Fig. 1:**
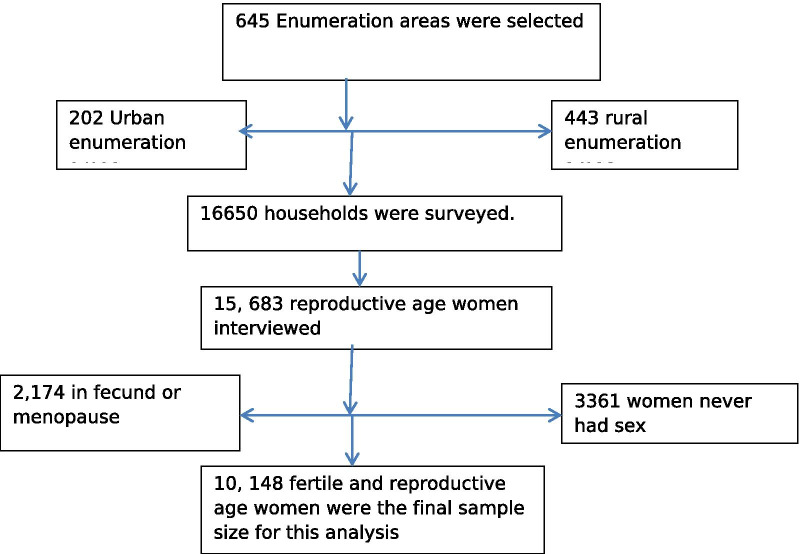
The flowchart for sampling and data extraction procedure, Ethiopian Demographic and Health Survey 2016. The horizontal arrow shows the excluded study participants from the surveyed households. More detail of the survey data collection method and study setting is available from EDHS 2016 report [12]

The EDHS 2016 is a fourth nationwide survey conducted in Ethiopia. There are nine regional states and two city administrations which are subdivided into 68 zones, 817 districts and 16,253 kebeles (lowest local administrative units of the country) in the administrative structures of the country [12]. The LACs methods were freely available without any fee for reproductive-age women, and they were provided in all public health facilities in Ethiopia. The survey data was accessed from the measure Demographic and Health Survey (https://dhsprogram.com/) after being registered as an authorized user. A total of 16,650 households were surveyed and 15,683 of them gave response. We used weighted sample size of 10,148 reproductive-age women for final analysis. The details of sampling and data extraction process is given below (Fig. 1).

The dependent variable of the study was long acting contraceptive use. The independent variables were socio-demographic and economic variables directly taken from the survey data (see Tables 2, 3). There are also some community level variables we computed from the existing dataset. See Table 1 for definitions of some variables.

**Table 1:** Measurement of some variables

Variable	Measurements/definitions
Long acting contraceptive users	Women who were used one of the long acting contraceptive methods: Intrauterine device, Implant and Female sterilization considered as LACs user
Distance to health facility	Distance to health facility was measured as yes/no. As distance to a health facility was a big problem or not based on respondents subjective response
Community level media Exposure	Aggregated at the cluster level. Those clusters with above the median of the population were exposed to family planning message on media were considered as high media exposure

### Data management and analysis

The data were checked for missing values and zero coordinates or twenty one clusters that had no coordinate’s data for spatial analysis were excluded for spatial analysis. To analyze spatial data, Geographical Information System (ArcGIS version 10.6) and Sat scan software were used. As per the recommendation of the survey report we weighed the maternal data using the maternal data weighting variable. Weighted sample sizes of 10,439 reproductive-age women were used in the final analysis. The weighed data was used for spatial and none-spatial data analysis. Stata 14 was used for none-spatial data cleaning, and analysis.

### Spatial analysis

The spatial autocorrelation (Global Moran’s I) statistics was used to evaluate whether the LACs distribution is random or not at the national level. Moran’s I value less than zero, equal to zero, and greater than zero indicates dispersed, random and clustered distribution of LACs respectively. A statistically significant Moran’s I (p < 0.05) leads to rejection of the null hypothesis and indicates the presence of spatial autocorrelation [23, 24].

Hot spot analysis was computed to measure how spatial autocorrelation varies over the study location by calculating Gi* statistic for each area. The Z-score, and p-values were computed to test the presence of significant clustering. If the z-score is between − 1.96 and + 1.96, the p-value would be larger than 0.05, and the pattern might be by chance. Statistical values with high Gi* indicate “hotspot” whereas low Gi* means a “cold spot” [23–25]. Spatial interpolation, using Kriging spatial interpolation method, was employed to predict the un-sampled from sampled measurements [26, 27].

Spatial scan statistical analysis was employed to test for the presence of purely spatial low LACs utilizing clusters using Kuldorff SaTScanTM version 9.6 software. The spatial scan statistics uses scanning window that moves across study area. Women who used LACs were taken as cases and women who did not use LACs as controls. We assumed that the dependent variable was binary, and Bernoulli assumption was considered. The default maximum spatial cluster size of < 50 of the population was used, as an upper limit, which allowed both small and large clusters to be detected and ignored clusters that contained more than the maximum limit be ignored. The primary and secondary clusters were identified and assigned p-values and ranked based on their likelihood ratio test, on the basis of 999 Monte Carlo replications [28].

### Statistical analysis of associated factors

Since the data have hierarchical nature, multilevel logistic regression was used to identify factors associated with LACs utilization at two levels: individual and community (cluster) levels. For this multilevel logistic regression analysis, four models were constructed. The first model was an empty model without any explanatory variables was meant to evaluate the extent of the cluster variation on LACs utilization. The second model was adjusted for individual level variable: the third model was for community level variables, while the fourth model was adjusted for both the individual and community level variables simultaneously. Variables with a p-value less than 0.2 in the Bi-variable analysis were used for multi-variable analysis. The fixed effect parameters Adjusted Odds Ratio (AOR) with 95% CI and a p-value < 0.05 was used to declare statistical significance.

Before interpreting multilevel logistic regression, model comparison was performed by using deviance and log likelihood ratio test. The random effects (variation of effects) were measured by intra cluster correlation coefficient (ICC), proportional change in variance (PCV), and Median odds ratio (MOR). The ICC explains the cluster variability [29] while PCV can measures the total variation due to factors at the community and individual level; MOR reflects the unexplained cluster heterogeneity and measures the area variance as odds ratios [30, 31]. The models with the lowest deviance, the highest PCV and the lowest MOR were selected as a better fitted model for interpretation. A multi-collinearity test was done to rule out a significant correlation between independent variables.

## Results

### Individual and community level characteristics of respondents:

More than half, 5982 (57.30%), and 6106 (58.49%) of the respondents had no formal education and wanted to have other children in the future respectively. Of the study participants, the majority, 9,110 (87.27%), were married (Table 2).

**Table 2:** Individual and house hold level characteristics of respondents in Ethiopia, 2016 (N = 10,439)

Variable	Frequency (N)	Percent (%)
Age of respondents		
15–24	2797	26.79
25–34	4682	44.86
35–49	2960	28.36
Working status of women		
Not working	6993	66.99
Working	3446	33.01
Marital status		
Never married	387	3.70
Married	9110	87.27
Formerly married	942	9.02
Wealth index		
Poorest	1985	19.02
Poorer	2024	19.39
Middle	2075	19.87
Richer	1976	18.93
Richest	2379	22.79
Educational status		
No education	5982	57.30
Primary	3150	30.17
Secondary and above	1307	12.52
Number of living children		
No children	1347	12.90
Have 1–2	3400	32.57
Have 3–4	2695	25.81
Have > 4	2997	28.71
Terminated pregnancy		
No	9432	90.35
Yes	1007	9.65
Visited HF in the last 12 month		
No	5226	50.06
Yes	5213	49.94
Fertility preference		
Want another	6106	58.49
Undecided	588	5.64
Want no more	3745	35.87
Age at first sex		
≤ 18	8054	77.15
> 18	2385	22.85
Age at first birth		
≤ 20	6772	73.90
> 20	2392	26.10
Media exposure		
No	7991	76.55
Yes	2448	23.45
Source of contraceptives		
Government	3225	30.94
Non-governmental organization	49	0.47
Private	595	5.71
Don’t know	6555	62.88
Distance to health facility		
Not a big problem	4924	47.21
A big problem	5510	52.79
Visited by HEW in the last 12 month		
No	7360	70.50
Yes	3079	29.50

From the study participants, majority of them, 8496 (81.38%), and 9345 (89.53%), were rural dwellers and from an agrarian regions respectively (Table 3).

**Table 3:** Community level characteristics’ of the respondents in Ethiopia, 2016 (N = 10,439)

Variable	Weighted frequency(N)	Weighted percent (%)
Residence		
Urban	1943	18.62
Rural	8496	81.38
Contextual region		
Agrarian	9345	89.53
Pastoral	522	5.00
Urban	572	5.47
Community level media exposure		
High media exposure	4358	41.75
Low media exposure	6081	58.25

### Spatial distribution of long acting contraceptive utilization among reproductive age women in Ethiopia

**Fig. 2:**
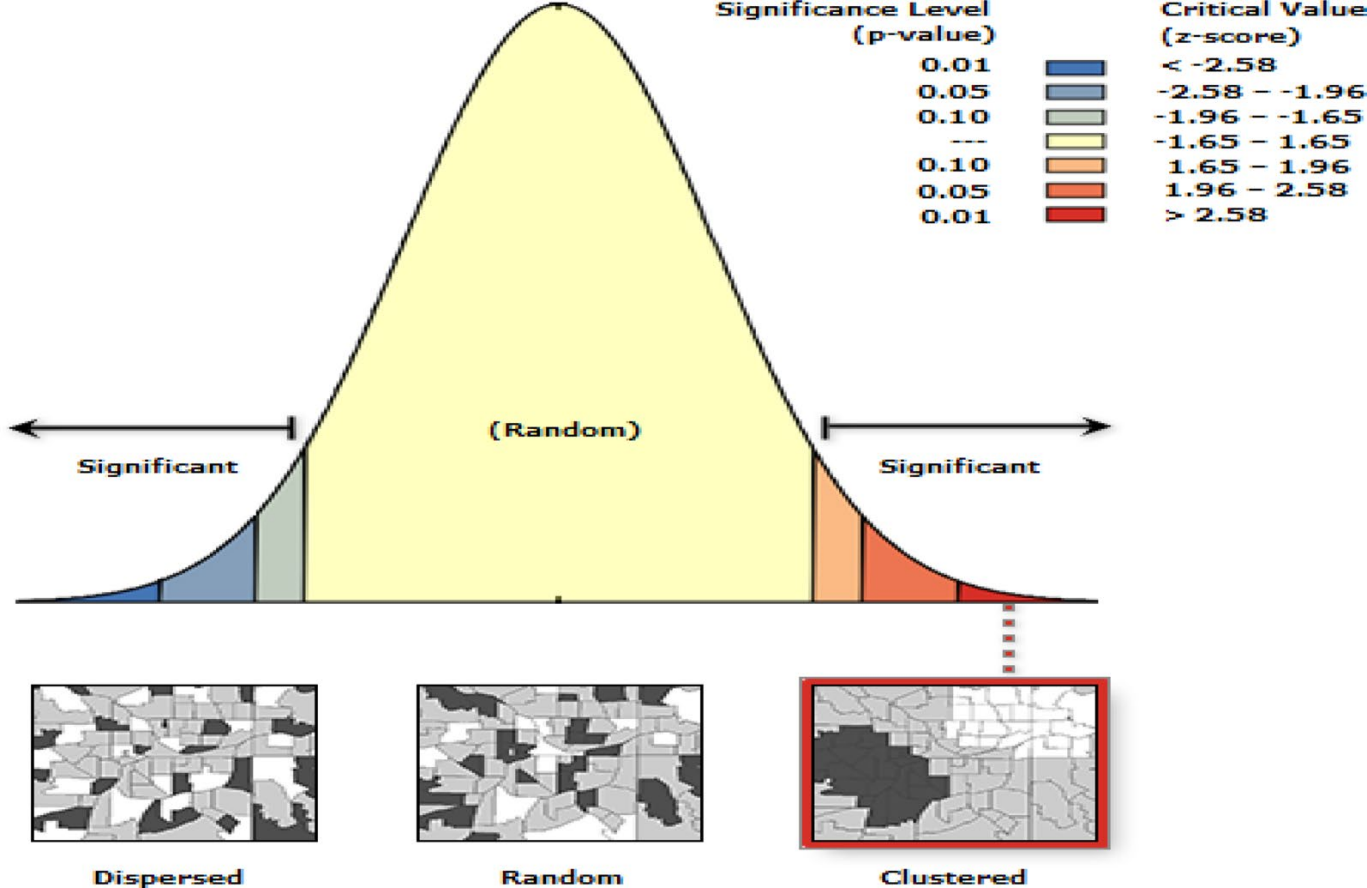
Spatial auto correlation analysis of long acting contraceptive utilization in Ethiopia, 2016

The spatial distribution of long acting contraceptive utilization was found to be non-random, Global Moran’s I 0.30 (p<0.01) (Fig. 2). This implies that further analysis is required to identify local level clusters.

**Fig. 3:**
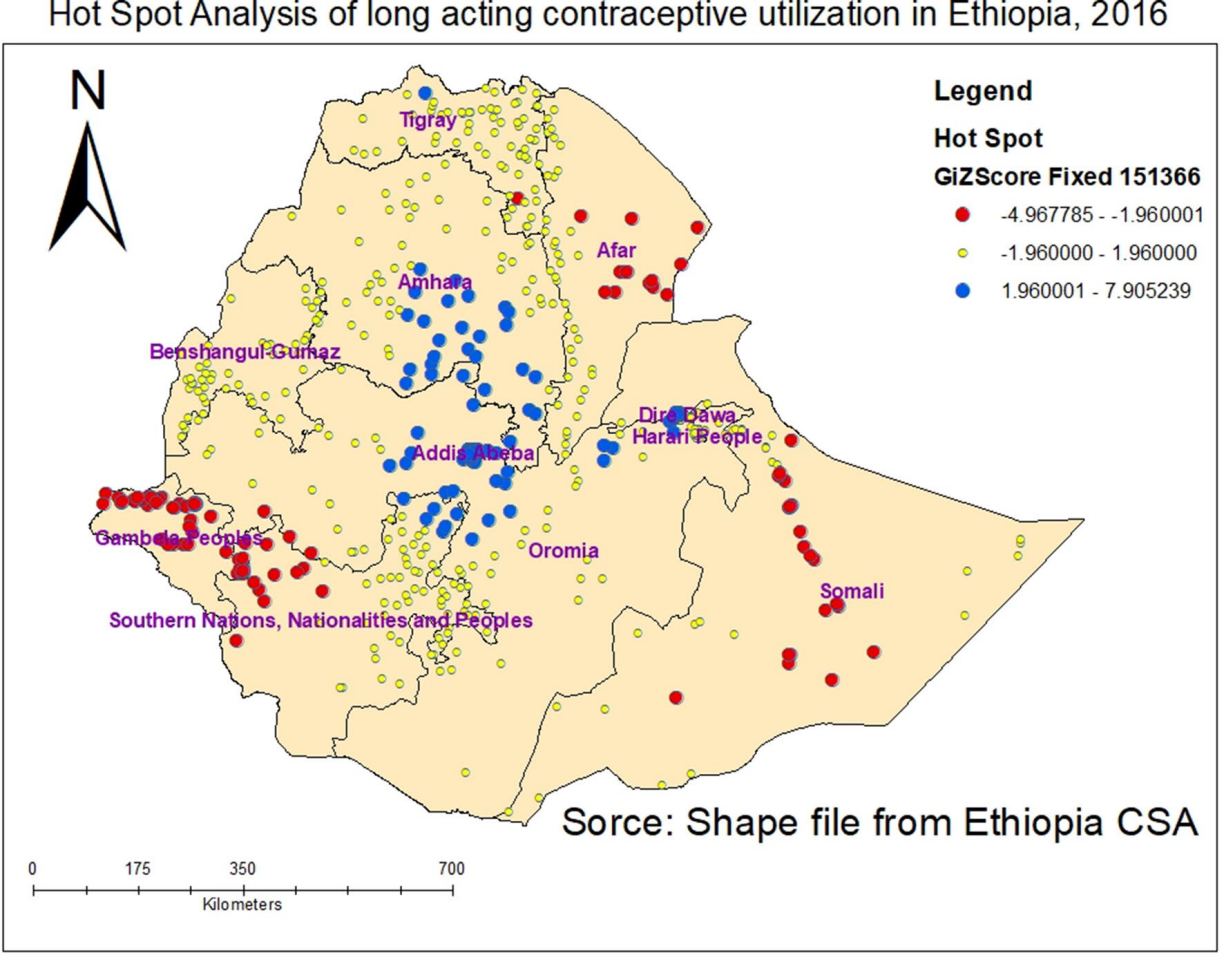
Hot Spot identification of long acting contraceptive utilization across regions in Ethiopia, 2016

Spots where there is high LACs utilization spots are represented by dark blue clustered points. These spots were observed in the central part of the country which encompasses Addis Ababa, Central and Eastern Oromia, Central and Southern part of Amhara, Northern part of SNNPR, Central Tigray, and Dire Dawa. The low LACs utilized spot were found in Somali, Afar, Gambela, northern part of SNNPR and Southeastern part of Tigray as represented by dark red colors (Fig. 3).

**Fig. 4:**
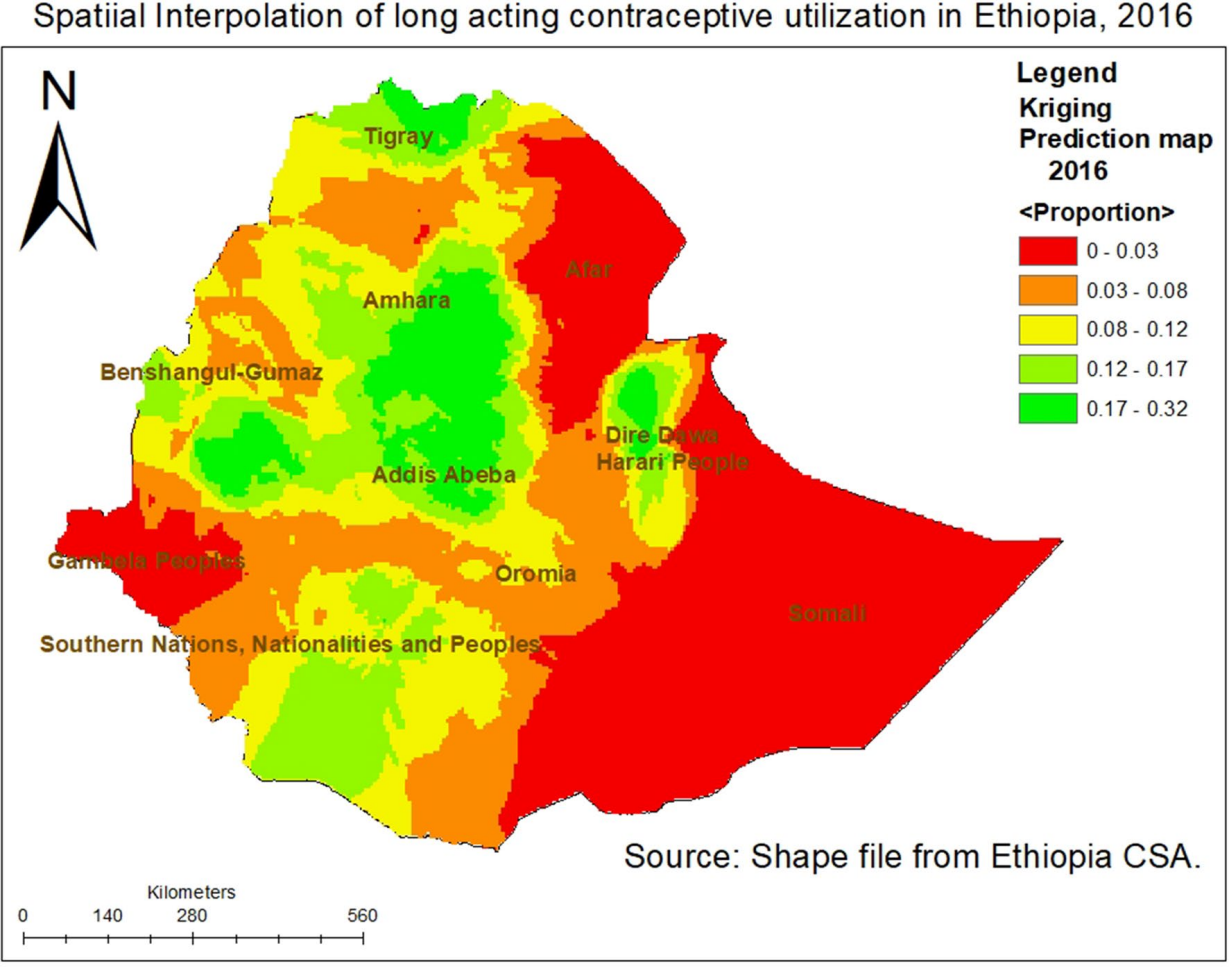
Kriging interpolation of LACs utilization across regions in Ethiopia, 2016

Using the ordinary kriging interpolation, the green ramp color on the map indicates the predicted highest LACs utilization rates in Amhara region, and Addis Ababa city administration. However, the red ramp color indicates low LACs utilized areas predicted in Somali, Afar, and Gambela (Fig. 4).

**Fig. 5:**
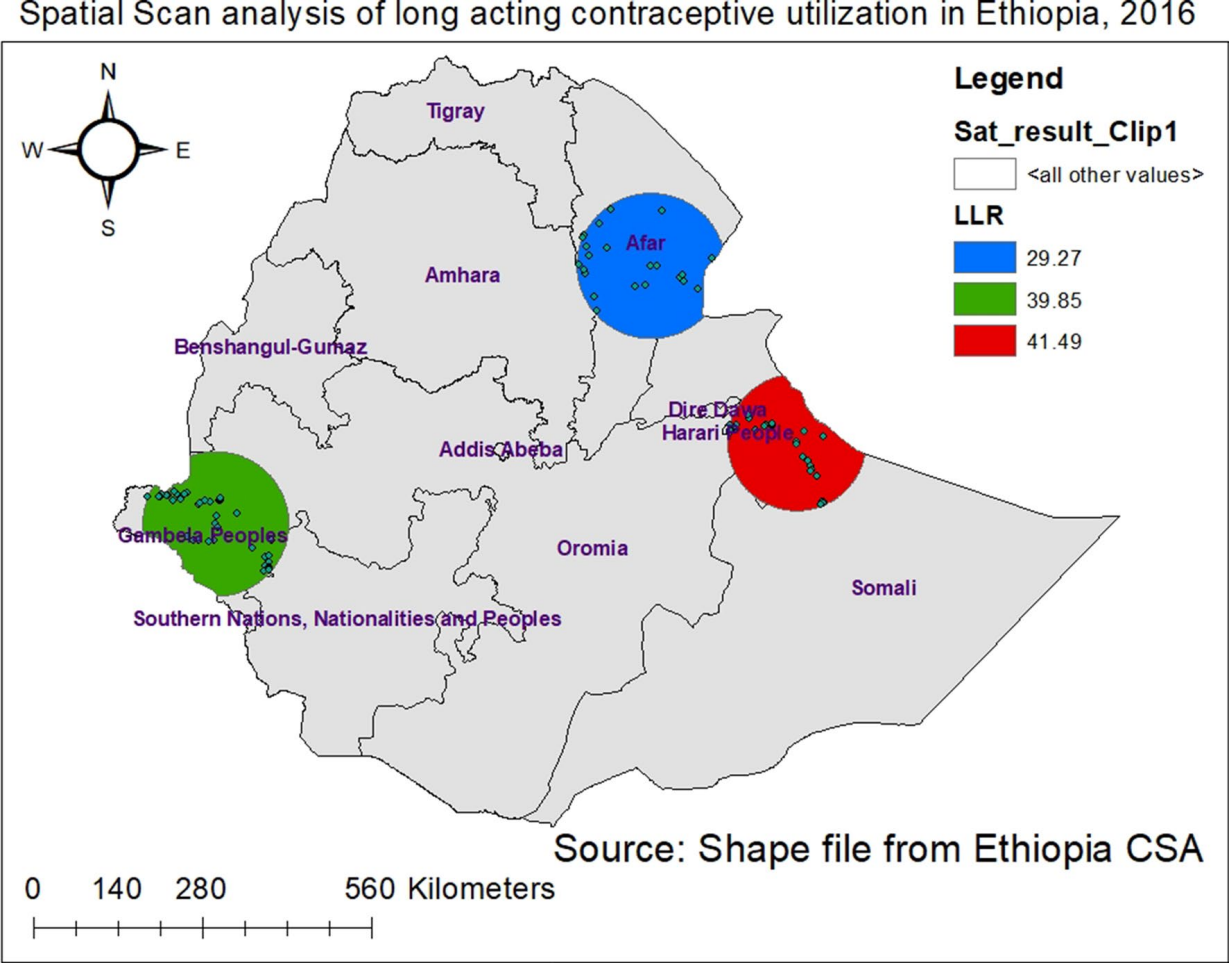
Spatial scan analysis to detect most likely clusters of long acting contraceptive utilization

The spatial scan statistics identified a total of 98 high and modest performing spatial clusters of LACs utilization. Of these, 29 clusters were most likely primary clusters (high performing clusters) accounting for 29.59%, and 69 were secondary clusters (modest performing clusters) accounting for 70.41%. The bright red colors (rings) indicate the most statistically significant spatial window which contains primary clusters located in the Somali and Harari people regions (Fig. 5). This was centered at 9.107168 N, 43.165844 E with 113.02 km radius, with a relative risk (RR) of 0.04 and Log-likelihood ratio (LLR) of 41.49, at p-value < 0.01. From the two secondary clusters, the first one is located in the Gambela, Western part of SNNPR and Western Oromia; the second one is located in the Afar region. The first secondary clusters spatial window was centered at 7.893414 N, 34.522102 E with 119.34 km radius, with a relative risk (RR) of 0.17 and Log-Likelihood ratio (LLR) of 39.85, at p-value < 0.01. The second secondary clusters spatial window was centered at 11.726887 N, 40.997478 E with 119.91 km radius, with a relative risk (RR) of 0.08 and Log-Likelihood ratio (LLR) of 29.27, at p-value < 0.01 (Fig. 5, Additional file 1).

### Associated factors of long acting contraceptive utilization

#### The random effect analysis

In Model 1, the empty model, 29.33% of the total variance of LACs utilization was accounted by between-cluster variation. Similarly, the between-cluster variability declined over the successive models, from 29.33% in the empty model, to 21.34% in the individual-level model, 20.30% in the community-level model, and 19.29% in the final model. In relation to ICC, the combined model with higher PCV; that is, 42.34% of variance in LACs utilization could be explained by the combined factors at the individual and community levels. The MOR for LACs utilization was 3.03 in the empty model, which implied the presence of variation between communities. The unexplained community variation in LACs utilization decreased to a MOR of 2.32 when all factors were added to the empty model. This indicates that even though individual and community level factors were considered, the clustering effect was significant. The deviance value was used to select the best fitting models among fitted two-level logistic regression models. The final model had lower deviance value which is 5709.08, this indicates that the final model was better fitted model in explaining LACs utilization as compared to other models.

#### The fixed effect analysis

The present study used a two-level mixed effects logistic regression model to analyze the effects of individual characteristics and community level factors affecting the utilization of long acting contraceptives.

In this study, the odds of utilizing long acting contraceptive methods among married women were 2.50 times (AOR = 2.50, 95% CI: 1.29–4.85) higher as compare to never married or single women. The odds of utilizing long acting contraceptive methods among currently working women were 1.33 times (AOR = 1.33, 95% CI: 1.07–1.65) higher as compared to women that are not working currently. The odds of long acting contraceptive utilization among women having one to two and three to four children were 2.13 (AOR = 2.13, 95% CI: 1.42–3.20), and 1.66 (AOR = 1.66, 95% CI: 1.01–2.73) times higher as compared to women having no alive children.

Regarding fertility preference, women who have not desire for more children were 1.42 times (AOR = 1.42, 95% CI: 1.09–1.84) more likely to use long acting contraceptive than women who have desire for other children. Women who had previous history of abortion had 44% (AOR = 0.56, 95% CI: 0.39–0.80) reduced odds of LACs utilization as compared to their counter parts. Women who said distance is a big problem had 24% (AOR = 0.76, 95% CI: 0.61–0.95) lower odds of LACS utilization as compared to women who said distance is not a big problem. Considering to the regional variation in the utilization of long acting contraceptive in Ethiopia, women’s residence in pastoral regions had strong negative effect on utilization of LACs, which reduced utilization of LACs by 79% (AOR = 0.21, 95% CI: 0.13–0.32) as compared to agrarian regions. Nevertheless, women living in urban regions had greater odds (AOR = 1.57, 95% CI: 1.15–2.15) for utilizing LACs compared with women living in agrarian regions (Table 4).

**Table 4:** Multilevel mixed effect logistic regression analysis of individual and community level factors associated with LACs utilization among reproductive age women in Ethiopia, 2016

Characteristics fixed effect	Model I	Model II AOR (95% CI)	Model III AOR (95% CI)	Model IV AOR (95% CI)
Age of respondent				
15–24		1		1
25–34		1.30 (0.98–1.75)		1.22 (0.91–1.63)
35–49		1.12 (0.80–1.58)		1.00 (0.70–1.42)
Marital status				
Never married		1		1
Married		2.19 (1.12–4.29)		2.50 (1.29–4.85)**
Formerly married		1.45 (0.66–3.19)		1.60 (0.73–3.51)
Wealth index				
Poorest		1		1
Poorer		1.48 (1.01–2.18)		1.34 (0.92–1.97)
Middle		1.47 (0.98–2.20)		1.30 (0.86–1.96)
Richer		1.33 (0.88–1.99)		1.14 (0.75–1.72)
Richest		1.66 (1.03–2.66)		1.06 (0.60–1.87)
Educational Status				
No education		1		1
Primary		0.80 (0.61–1.05)		0.76 (0.58–1.00)
Secondary and above		1.00 (0.68–1.47)		0.88 (1.09–1.84)
Working status				
Not working		1		1
Working		1.36 (1.09–1.69)		1.33(1.07–1.65)*
Number of living children				
Have no children		1		1
Have 1–2		2.11 (1.41–3.17)		2.13 (1.42–3.20)**
Have 3–4		1.60 (0.98–2.62)		1.66 (1.01–2.73)*
Have > 4		1.45 (0.83–2.53)		1.62 (0.92–2.85)
Fertility preference				
Want another		1		1
Undecided		0.81 (0.48–1.37)		0.80 (0.48–1.35)
Want no more		1.47 (1.14–1.91)		1.42 (1.09–1.84)*
Terminated pregnancy				
No		1		1
Yes		0.57 (0.40–0.82)		0.56 (0.39–0.80)**
Distance to HF				
Not a big problem		1		1
A big problem		0.72 (0.58–0.90)		0.76 (0.61–0.95)*
Residence				
Urban			1	1
Rural			0.71 (0.47–1.08)	0.65 (0.36–1.19)
Community media exposure				
High media exposure			1	1
Low media exposure			0.73 (0.49–1.07)	0.74(0.50–2.15)
Contextual region				
Agrarian			1	1
Pastoral			0.19 (0.12–0.28)	0.21 (0.13–0.32)**
Urban			1.33 (0.98–1.81)	1.57 (1.15–2.15)**
Random effect	Model I	Model II	Model III	Model IV
Variance	1.37	0.89	0.84	**0.79**
ICC (%)	29.33	21.34	20.30	**19.29**
PCV (%)	Reference	35.04	38.69	**42.34**
MOR	3.03 (2.69–3.49)	2.45 (2.20–2.77)	2.39 (2.14–2.69)	**2.32 (2.10–2.61)**
LLR	− 3070.92	-2917.21	− 2961.30	**− 2854.54**
Deviance	6141.84	5834.42	5922.60	**5709.08**

Bold indicates the improvement of model diagnosis parametrs in the final model as compared to other models

*AOR* adjusted odds ratio, *CI* confidence interval, *1* Reference

*Significance at P < 0.05, **Significance at P < 0.01

Model I: null model, model II: individual level variables, model III: community level variables, model IV: individual and community level variables

## Discussion

Clusters where there is low LACs utilization were found in Somali, Afar, and Gambela regions. This could be due to the relative under development and low urbanization of these areas, which may have contributed to the low utilization of long acting contraceptive [32]. The other possible justification might be the low availability of health facilities [33], and trained health professionals on family planning. Considering the health personnel’s availability, the 29, 26 and 27% of the health facilities in Afar, Somali and Gambela regions had at least one trained staff on family planning while more than 55% of the health facilities in other regions had a trained health professionals [34]. Which implied the lack of trained health personnel in these regions may contribute for low utilization of LACs.

Regarding predictors of long acting contraceptive utilization, regression analysis identified several individual and community level factors that influenced utilization of long acting contraceptive. Marital status, working status of women, number of living children, fertility preference, terminated pregnancy, distance to health facility, found in the urban and pastoral areas of contextual region were among factors significantly associated with long acting contraceptive utilization among reproductive-age women in Ethiopia.

In this study, marital status was found to be significantly associated with long acting contraceptive utilization. Married women were 2.50 times more likely to use long acting contraceptive compared to those women’s who are never married. This finding is consistent with previous studies done in Ethiopia [8, 35], Nigeria [36], and a study done by using DHS data of 18 Sub-Saharan countries [37]. The possible reason might be that married women have regular sexual practice with their partners. According to the later study, most married women (91%) had at least one and more children; this may increase their desire to space and limit pregnancy.

Working status of women was also significantly associated with the utilization of LACs among reproductive age women. Currently working women were 1.33 times more likely to use LACs than those who were not working. This is supported by findings of other studies conducted in Ethiopia where currently working women were more likely to use the LACs [17, 35]. The possible reason might be that the employed women had better knowledge about contraceptive and they cope with traditional culture and belief which may obstruct LACs utilization [17].

Long acting contraceptive utilization of women who have one to two, and three to four living children had 2.13, and 1.66 times higher odds of LACs utilization respectively as compared to women who have no living children. This result is supported by findings from studies done in Ethiopia [17], Malawi [18], Ghana [38] and Iran [21]. The possible reason might be that women who achieved the desired number of children want to space or limit further pregnancies by using LACs [6].

This study found a significant relationship between fertility preference and LACs utilization. Women who had no desire for more children were 1.42 times more likely to utilize LACs than those women who want other children. This finding is concurrent with studies conducted in different parts of Ethiopia [17, 39–42] and Rwanda [43]. The possible explanation could be that women who have no desire for extra children use LACs method to achieve their desires. Since long acting and permanent contraceptive method is safe, cheap and long term in preventing unwanted pregnancy, it is a method of choice by couples who needs to completely delay childbirth [2].

Women who had ever terminated pregnancy had 44% lower odds of LACs utilization than their counterparts. This might be due to the fact that women who had terminated pregnancy reduces the utilization of LACs because of intention to give birth. However, a study done in Ethiopia [44], showed that women who ever had a terminated pregnancy were more likely to utilize LACs than their counterparts.

In this study, women who reported distance to health facility as a big problem had 24% lower odds of LACs utilization as compared to those reported distance to health facility is not a big problem. This study is supported by findings from other study conducted in Ethiopia where living in proximate to health facilities increase the utilization of modern contraceptives including LACs [45]. The possible explanation might be that lack of transportation and access of health facilities may contribute to the low utilization of LACs and other maternity continuum of cares [45, 46]. This finding is in contrary to findings of a study done in Adama town, Ethiopia [15]. This discrepancy could be due to population difference; the study conducted in Adama was on urban population whereas more than 80% of the study population included in the national survey of Ethiopia includes more than 80% of the study population were rural dwellers. These urban dwellers had no distance problem to arrive at health facilities.

### Strength of the study

Using a national representative data might helped us to have better estimation of parameters. Equally, applying spatial analysis was important to identify the geographic variation of LACs utilization. Thus, this study will help the policy makers to design or strengthen intervention strategies based on the identified geographic variations.

### Limitation of the study

We are confident that our study is strong but it is not immune to limitations. As we used secondary data analysis, we could not access important variables such as behavioral factors, health service qualities and some uncertainties on the geographic coordinate data.

## Conclusion

This study indicated a considerable geographic difference in long acting contraceptive utilization across regions of Ethiopia. Clusters where there is statistically significant low long acting contraceptive utilization were detected in Afar, Somali and Gambela regions. Being married, want no more children, having one to two and three to four children, found in urban areas, and working status of women increased the likelihood of long acting contraceptive use while having previous history of abortion, far from health facility, and living in the pastoral areas reduced the likelihood of utilization of long acting contraceptives.

## Supplementary Information

**Additional file 1.** Most likely cluster numbers of low utilization of long acting contraceptives in Ethiopia detected by spatial Scan statistics, EDHS 2016.

## Data Availability

The data used for preparation of this manuscript are available from http://www.dhsprogram.com and anyone can access through online request as authorized user. The authors prepared the data that was used for preparation of this manuscript can be shared if required.

## Abbreviations

AOR: Adjusted odds ratio
EDHS: Ethiopian demographic and health survey
EA: Enumeration area
LACs: Long acting contraceptives
LLR: Log likelihood ratio
RR: Relative risk
