# Supplementary material for: Geographic variation and associated factors of long-acting contraceptive use among reproductive-age women in Ethiopia: a multi-level and spatial analysis of Ethiopian Demographic and Health Survey 2016 data
Source: Reprod Health. 2021 Jun 10;18:122. doi: 10.1186/s12978-021-01171-2 (PMC8194103; doi:10.1186/s12978-021-01171-2)
Supplement: Supplementary file 1 — Additional file 1. Most likely cluster numbers of low utilization of long acting contraceptives in Ethiopia detected by spatial Scan statistics, EDHS 2016. [file 12978_2021_1171_MOESM1_ESM.docx]

Additional file 1: Most likely cluster numbers of low utilization of long acting contraceptives in Ethiopia detected by spatial Scan statistics, EDHS 2016.

| Cluster | Enumeration areas (detected) clusters | Coordinates/ Radius | Population | Cases | RR | LLR | P-value |
| --- | --- | --- | --- | --- | --- | --- | --- |
| 1 | 77, 568, 527, 22, 116, 33, 239, 64, 439, 57, 573, 251, 210, 8, 214,186, 566, 1, 622, 436, 212, 458, 553, 588, 501, 454, 521, 68, 357 | (9.107168 N, 43.165844 E) / 113.02 km | 434 | 2 | 0.04 | 41.49 | <0.01 |
| 2 | 593, 219, 265, 446, 270, 106, 231, 221, 114, 448, 284, 469, 291, 47, 549, 567, 343, 105, 63, 417, 13, 315, 603, 346, 326, 233, 426, 69, 260, 104, 119, 46, 592, 507, 554, 370, 526, 299, 197, 243, 459, 465, 552, 168, 371, 536 | (7.893414 N, 34.522102 E) / 119.34 km | 702 | 13 | 0.17 | 39.85 | <0.01 |
| 3 | 205, 499, 178, 334, 570, 440, 632, 596, 348, 75, 427, 389, 4, 368,544, 191, 571, 241, 599, 55, 189, 344, 332 | (11.726887 N, 40.997478 E) / 119.91 km | 334 | 3 | 0.08 | 29.27 | <0.01 |
